# Conformal metasurface-coated dielectric waveguides for highly confined broadband optical activity with simultaneous low-visibility and reduced crosstalk

**DOI:** 10.1038/s41467-017-00391-0

**Published:** 2017-08-25

**Authors:** Zhi Hao Jiang, Lei Kang, Douglas H. Werner

**Affiliations:** 1 Department of Electrical Engineering, The Pennsylvania State University, University Park, PA 16802 USA; 2 State Key Laboratory of Millimeter Waves, School of Information Science and Engineering, Southeast University, Nanjing, 210096 China

## Abstract

The ability to achieve simultaneous control over the various electromagnetic properties of dielectric waveguides, including mode confinement, polarization, scattering signature, and crosstalk, which are critical to system miniaturization, diversity in functionality, and non-invasive integration, has been a highly sought after yet elusive goal. Currently existing methods, which rely on three-dimensional artificial cores or claddings and/or structural chirality, provide efficient paths for obtaining either highly confined modes, optical activity, or a low-scattering signature, but at the expense of increased propagation loss, form factor and weight. Here, by tailoring the unique anisotropy and exploiting the inter-cell coupling of metasurface coatings, we report a unified approach for simultaneously controlling the diverse optical properties of dielectric waveguides. The experimentally demonstrated highly confined sub-wavelength dielectric waveguide with a low-visibility and broadband optical activity represents a transformative wave manipulation capability with far reaching implications, offering new pathways for future miniaturization of dielectric waveguide-based systems with simultaneous polarization and scattering control.

## Abstract (web summary)

Controlling all the optical properties of dielectric waveguides is a challenging task and often requires complicated core- and cladding designs. Here, Jiang et al. demonstrate that a thin metasurface coating can control several optical properties simultaneously over a broad frequency range.

## Introduction

Conventional electromagnetic devices have typically relied on tailoring the geometrical shape and structural arrangement of natural materials, e.g., lenses and gratings, which usually possess bulky form factors, as well as lack functional versatility (a direct consequence of the limited optical response offered by available constitutive materials). In contrast, artificially structured materials, also referred to as metamaterials, exhibit significantly expanded electromagnetic response diversity owing to their rich optical properties that can be engineered on a subwavelength scale, but they still suffer from undesirably bulky volumes and/or associated high absorption loss. Recent advances in metasurfaces^1–3^, a class of quasi-two-dimensional (quasi-2D) metamaterials comprised of deep sub-wavelength resonators, have opened up a path towards the comprehensive molding of electromagnetic waves based on a low-loss, fully-planarized, and conformal platform. By designing the sub-wavelength building blocks of a metasurface, the superficial electromagnetic properties, often represented by a spatially and spectrally dispersive surface polarizability^4^ or surface impedance tensor^5, 6^, can be tailored almost at will. Through this approach, the flow of electromagnetic waves can be manipulated with an abundant degree of flexibility, as demonstrated by devices which exhibit anomalous reflection and refraction^7, 8^, broadband polarization control^9–11^, wavefront conversion^12–14^, diffusion-like scattering^15^, electromagnetic cloaking and illusion^16–18^, spin-Hall effect of light^19^, and many other novel properties.

As one of the most fundamental components for routing and transferring electromagnetic signals, classical dielectric waveguides (DWs) have been widely investigated over the last century^20, 21^ for applications ranging from microwave/millimeter-wave integrated circuits^22^ and antenna systems^23^ to electro-optical sensors^24^ and optical communication systems^25^. By virtue of the inherent non-cutoff fundamental guided mode, DWs offer a single-mode operation over a very broad bandwidth with extremely low propagation loss, as compared with fully enclosed hollow metallic waveguides and metallic surface plasmon polariton (SPP) waveguides^26^. The main shortcoming of conventional DWs, especially those comprised of light-weight materials with a low dielectric constant, is the poor power confinement. This can cause significant crosstalk and interference between neighboring DWs, thereby hampering their deployment in a densely packed layout for effective system integration and miniaturization. Hence, extensive efforts have been carried out with the aim of achieving a highly confined mode within the DWs, while simultaneously maintaining a low-loss single-mode operation.

The early pioneering works tackling this issue used structured claddings outside the core to prevent leakage of the guided wave, including Bragg reflectors^27^, totally reflecting coatings^28^, and photonic band gap structures^29^. Recently, anisotropic artificial claddings with an extraordinary hyperbolic dispersion, realized by concentric alternating metal and dielectric layers^30^ and multi-layered nanowire arrays^31^, have been introduced in order to enable sub-wavelength mode confinement, which share conceptual similarities with grooved circular waveguides that can support hybrid mode propagation^32^. An alternative approach has been to engineer the core in order to achieve a stronger mode confinement by, for example, using a cylindrically multi-layered structure with a near-infinite radial component of the refractive index^33^ or employing parity-time symmetric epsilon-near-zero bi-layer slabs^34^. These two methods, which often require bulky metallo-dielectric structures with extreme material parameters, unfortunately limit the operation to a narrow frequency range and increase the volume, weight and cost of the waveguides. More recently, a third approach has been demonstrated that allows hybrid guided modes to be supported at a sub-wavelength scale by properly merging dielectric cores and metallic plasmonic structures into a single heterogeneous waveguide^35–37^. However, the added three-dimensional (3D) metallic plasmonic structures inevitably increase the profile and degrade the polarization purity of the guided mode as compared to those in the original DWs and suffer from an increased propagation loss.

In addition, methods to control the other inherent properties of a DW, such as the scattering signature and mode polarization, have also been actively explored. For non-invasive applications, invisible DWs have been theoretically considered by embedding 3D metallic nanowire clusters into the dielectric core^38^. Optically active DWs have received considerable attention due to their inherent optical chirality, one of the most interesting and useful properties governing wave–matter interactions, that can be exploited for facilitating a variety of transformative communication, sensing and imaging devices^39, 40^. So far, two strategies have been pursued to integrate optical activity into DWs. The first one concerns embedding low-loss chiral materials into the core to form a chirowaveguide^41–43^, which unfortunately has turned out to be rather challenging owing to the difficulty in realizing low-loss isotropic 3D chiral media. The other approach involves introducing 3D structural chirality into a locally non-chiral medium^44, 45^, such as twisting a linearly birefringent fiber^46^, helically winding a circular DW^47^, twisting photonic crystal fibers with a noncircular guiding core^48^, and introducing multihelical refractive index profiles into the core material^49^.

Thus far, a simple and efficient solution for versatile control over the diverse electromagnetic properties of DWs has yet to be achieved. Here, we propose a conformal metasurface-enabled approach which allows for simultaneous manipulation of the mode confinement, polarization and scattering signature of a DW while avoiding the increased volume, weight and loss typically associated with previous approaches that exploit 3D structures. The demonstrated conformal coating, which contains two concentric quasi-2D tensorial metasurface layers—an inner guiding layer and an outer cloaking layer—enables a highly confined sub-wavelength DW with a low-visibility and broadband optical activity. Moreover, we show that the sub-wavelength guiding property is well maintained under structural bending by locally matching the dispersion of the unit cells of the metasurface guiding layer. The devices were fabricated and the measured results are in strong agreement with theoretical/numerical predictions, highlighting the fidelity and robustness of the proposed technology. The integrated open waveguide holds a great promise for future system miniaturization with simultaneous polarization and scattering manipulation. Because of its non-magnetic properties, the unified theory and design approach proposed here can be extended to achieve versatile control of the properties of DWs in the terahertz and possibly even infrared regimes.

## Results

### Dielectric waveguides coated by a metasurface

**Fig. 1:**
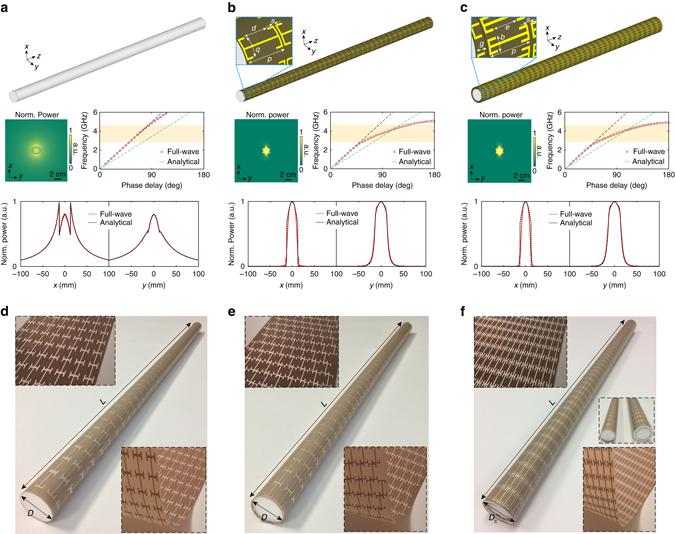
A sub-wavelength dielectric rod waveguide coated by conformal metasurface layers. Configuration, dispersion curves, normalized power distribution in the *x*-*y* plane at 3.4 GHz, and normalized power cuts along the *x*- and *y*-axis at 3.4 GHz of **a** a bare Teflon rod waveguide with *ε*_r_ = 2.1, **b** the same Teflon rod waveguide coated by the metasurface guiding layer, and **c** the same Teflon rod waveguide coated by the metasurface guiding and cloaking layers. For the dispersion curves, the *dashed gray* and *dashed green lines* are the light lines of free space and Teflon, respectively. The highlighted region is from 2.8 to 4.59 GHz. The photographs of the fabricated and assembled samples are shown in **d** for the Teflon rod coated by the metasurface guiding layer, **e** for the Teflon rod coated by the metasurface guiding layer with twisting perturbation, and **f** for the Teflon rod coated by the metasurface guiding and cloaking layers. The insets of **d**–**f** show enlarged views of the ultrathin flexible metasurfaces. The dimensions are: *b* = 3.9, *d* = 8.5, *D* = 25.4, *D*_*c*_ = 33.4, *e* = 8.5, *g* = 4, *L* = 600, *p* = 15, *s* = 0.5, all in millimeters. The substrate material is Taconic TLY-5 (*ε*_r_ = 2.2, tan*δ* = 0.0009) with a thickness of 0.1 mm

Among the supported modes in a circular dielectric rod waveguide, the hybrid HE_11_ mode, which is linearly polarized and has no cutoff, is the most interesting and widely used in practical applications. Here, without loss of generality, the HE_11_ mode is assumed to be propagating along the *z* direction (the axis of the rod) with the electric field linearly polarized along the *x* direction. The dispersion curve of the HE_11_ mode for the bare Teflon rod, with a sub-wavelength radius, follows the light line of free space very closely (Fig. 1a), owing to its corresponding weakly bounded mode profile. As shown by the power distribution in the transverse plane, i.e., the *x*–*y* plane, a significant amount of electromagnetic power resides in the region outside the core, which has a radius of around 0.144*λ*_0_ (where *λ*_0_ corresponds to the wavelength in free space at 3.4 GHz). Quantitatively, the mode area, defined by the cross sectional area that encompasses 90% of the total power associated with a particular mode, in this case is $39.4\lambda _0^2$. To achieve a stronger field confinement, dielectric materials with a significantly higher dielectric constant can be used, but only at the expense of a worsening impedance mismatch, as well as an increase in weight, loss, and cost.

Here, we propose a low-profile and light-weight solution by tailoring the electromagnetic properties of the boundary at the interface between the dielectric rod and free space, such that the characteristics of the guided modes can be controlled. The required boundary manipulation was achieved by a non-magnetic conformal impedance surface coating with a non-vanishing in-plane electromagnetic response (Fig. 1b), which relates the tangential electric ([*E*_φ_, *E*_z_]^T^) and magnetic fields ([*H*_φ_, *H*_z_]^T^) via a surface impedance tensor with non-zero values only for the off-diagonal elements as^5, 6^

1 $$\left[ {\begin{array}{*{20}{c}}{{E_{\rm{\varphi }}}} \\ {{E_{\rm{z}}}} \\ \end{array}} \right] = {{\bf{Z}}_{\rm{s}}}\left[ {\begin{array}{*{20}{c}}{{H_{\rm{\varphi }}}} \\ {{H_{\rm{z}}}} \\ \end{array}} \right] = \left[ {\begin{array}{*{20}{c}} 0 & {{Z_{{\rm{s\varphi z}}}}} \\ {{Z_{{\rm{sz\varphi }}}}} & 0 \\ \end{array}} \right]\left[ {\begin{array}{*{20}{c}}{{H_{\rm{\varphi }}}} \\ {{H_{\rm{z}}}} \\ \end{array}} \right].$$

By solving the associated eigenmode problem, the dispersion and field distribution of the HE_11_ mode can be obtained for a dielectric rod coated by a homogeneous tensorial impedance surface with different values of *Z*_sφz_ and *Z*_szφ_ (Supplementary Note 1). When *Z*_sφz_ = 0, i.e., the *φ* component of the electric field is forced to zero at the boundary, only an inductive *Z*_szφ_ allows for HE_11_ mode propagation. As a result, however, most of the power is located in the air region outside the central rod close to the coating surface, resembling the mode pattern of a strongly confined surface mode. In contrast, when *Z*_sφz_ = ±∞, i.e., an open circuit where the *z* component of the magnetic field is continuous across the boundary, as the value of *Z*_szφ_ transitions from capacitive to inductive regions, the giant anisotropy of the metasurface coating enables a strongly confined HE_11_ mode (Supplementary Note 2 and Supplementary Fig. 2). It was further found that the open circuit condition for *Z*_sφz_ can be relaxed, thereby implying that such a physical phenomenon can be preserved within a broad frequency range. Considering a specific case where *Z*_sφz_ = −1460*j* and *Z*_szφ_ = −26*j*, as the power distribution in the *x*–*y* plane at 3.4 GHz shows (Fig. 1b), a strong power confinement within the rod corresponding to a mode area of only 0.18$\lambda _0^2$ is observed, which is about 0.5% of that required by the conventional bare Teflon rod waveguide. In contrast to the bare DW, a sharp roll-off occurs at the surface of the Teflon rod as displayed in the normalized power cuts along the *x* and *y* axes, due to the tangential field discontinuity caused by the presence of the coating.

To implement the desired tensorial surface impedance at microwave frequencies, a metasurface guiding layer comprised of an array of capacitively coupled end-loaded dipoles^50^ with a sub-wavelength periodicity of 15 mm (~ *λ*_0_/6) printed on a Taconic TLY-5 substrate was employed. The substrate layer is ultrathin, i.e., with a thickness of only 0.1 mm (~ *λ*_0_/1000) and exhibits an extremely low loss (Supplementary Fig. 4). As shown in Fig. 1b, with the structured metasurface, the dispersion curve of the HE_11_ mode of the coated Teflon rod waveguide has a line-shape resembling that of a surface wave^26^, with a band edge at 5.04 GHz. It should be noted that at frequencies below 4.12 GHz, where the dispersion curve of the HE_11_ mode of the coated waveguide is located within the region bounded by the light lines of free space and Teflon, the radial wave number maintains a real value, meaning that the field is decaying only outside the rod. This behavior is distinct from the spoof SPP waveguides based on grooved metallic structures for which the fields are confined near the surface^51^. Here, the proposed coating retains the bulk HE_11_ mode but with a tight confinement. At higher frequencies, the field decays on both sides of the interface between Teflon and free space, i.e., the surface bounding mode now comes into play. However, due to the sub-wavelength radius of the rod and the smaller decaying factor within the rod as compared to that on the free-space side, at frequencies below 4.59 GHz (the upper bound of the highlighted region), the field distribution within the rod will remain relatively uniform without a null formed at the center. Even at a low frequency of around 2.8 GHz (the lower bound of highlighted region), the mode area is only 1$\lambda _0^2$, which is several orders smaller than that of the HE_11_ mode in the bare dielectric rod. The normalized power cuts along the *x* and *y* axes, as well as the dispersion curve calculated analytically using the effective surface impedance of the realistic metasurface (Supplementary Fig. 4) agrees well with those obtained by a full-wave electromagnetic solver^52^ (Fig. 1b) with only minor discrepancies at frequencies near the band edge. This confirms that the discrete metasurface well approximates a homogeneous tensorial impedance surface in the majority of the frequency range of interest, i.e., the highlighted region. Further analysis shows that the attenuation constant *α*_z_ *p* is smaller than 1.4 × 10^−3^, implying a propagation length of more than 82*λ*_0_ throughout the highlighted frequency range of interest (see Supplementary Note 3 and Supplementary Fig. 5) with a value reaching 424*λ*_0_ at 3.4 GHz. Even at terahertz frequencies, such a coating can still provide confined HE_11_ mode guidance with a low loss (see Supplementary Note 4 and Supplementary Fig. 6), while it should be recognized that other technological challenges need to be overcome for a successful translation of this technology into the terahertz regime. It should be noted that anisotropic impedance surfaces, implemented by transverse corrugated structures with a quarter-wavelength depth for satisfying the soft surface condition^53^, have previously been utilized to enable a Gaussian-like HE_11_ mode propagation inside an overmoded hollow metallic circular waveguide^32^, however, at the expense of a radius much larger than a wavelength and a greatly increased weight.

**Fig. 2:**
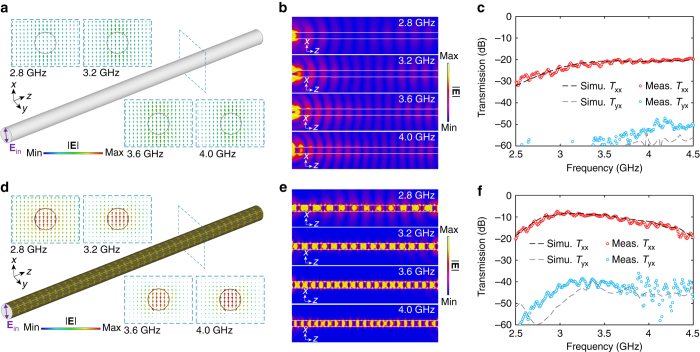
A sub-wavelength dielectric rod waveguide with a single-layer metasurface coating. Snapshots of the vector electric field distribution in the *x*-*y* plane at 2.8, 3.2, 3.6, and 4.0 GHz for **a** the bare Teflon rod waveguide and **d** the Teflon rod waveguide coated by the metasurface guiding layer when excited by an *x* polarized probe. Snapshots of electric field distribution in the *x*-*z* plane at 2.8, 3.2, 3.6, and 4.0 GHz for **b** the bare Teflon rod waveguide and **e** the Teflon rod waveguide coated by the metasurface guiding layer. Measured and simulated co-polarized (*T*_xx_) and cross-polarized (*T*_yx_) transmission between the transmitting and receiving probes for **c** the bare Teflon rod waveguide and **f** the Teflon rod waveguide coated by the metasurface guiding layer

On the basis of the dispersion properties of the infinitely-long rod, the coated DW with a finite length of 600 mm, i.e., ~ 6.8*λ*_0_ at 3.4 GHz, was then considered, where two small linearly polarized dipole probes were used to characterize the transmission. The simulated electric field distributions in the *x*–*y* plane at different frequencies are displayed in Fig. 2a, d. It can be observed that, when the metasurface-enabled guiding coating is included, significant enhancement in the power confinement is achieved over a broad frequency range with a bandwidth of more than 35%. The electric field distributions in the *x*–*z* plane for the cases without and with the coating are displayed in Fig. 2b, e, respectively. Throughout this broad frequency range, the electric field becomes strongly confined within the rod owing to the presence of the metasurface coating. Such a strongly confined mode implies the possibility of obtaining a subwavelength and lightweight low index waveguide with greatly reduced crosstalk. The flexible metasurface guiding layer, which contains 40 unit cells along the *z* direction and 8 unit cells in the *φ* direction, was fabricated and conformed to the Teflon rod (Fig. 1d). The measured co-polarized (*T*_xx_) and cross-polarized (*T*_yx_) transmission curves are presented in Fig. 2c, f, which demonstrate good agreement with the simulations. Compared with the bare Teflon waveguide, the proposed coating facilitates a 13–18 dB transmission enhancement in the majority of the frequency range of interest. The corresponding level of *T*_yx_ is about 30 dB lower than that of *T*_xx_ (Fig. 2f), indicating that the guided wave is linearly polarized with a well-preserved plane of polarization.

### Twisting metasurface enabled broadband optical activity

**Fig. 3:**
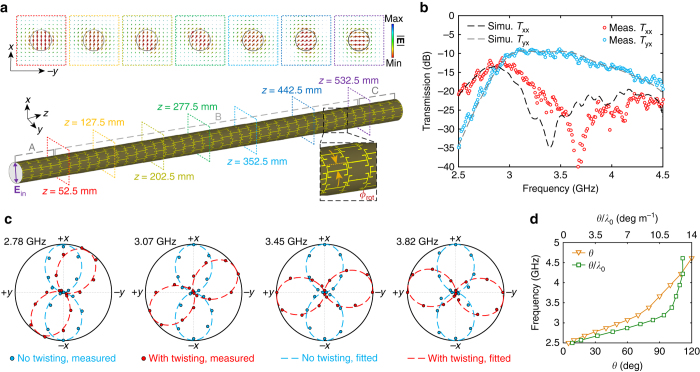
A sub-wavelength dielectric waveguide coated by a single-layer twisting metasurface. **a** Configuration and snapshots of the vector electric field distribution at 3.45 GHz in the *x*-*y* plane at seven different positions along the axis of the rod when excited by an *x* polarized probe. Sections A and C contain unit cells near the two ends, while section B contains unit cells in the center. **b** Measured and simulated co-polarized (*T*_xx_) and cross-polarized (*T*_yx_) transmission between the transmitting and receiving probes. **c** Measured state-of-polarization patterns of the Teflon rod waveguide coated by the metasurface guiding layer with and without twisting perturbation at different frequencies. **d** Measured rotation angle and the rotation angle per wavelength as a function of frequency

Here we show that, based on the highly confined modes enabled by the metasurface guiding layer, it is possible to achieve broadband optical activity within a sub-wavelength waveguide by incorporating structural chirality. Specifically, instead of twisting the entire waveguide around a virtual external axis^45–47^, only the discrete unit cells of the quasi-2D metasurface guiding layer were twisted to form an unconnected multifilar helical arrangement such that the coating remains conformal to the central rod. To do so, as shown in Fig. 3a, the coated DW was divided into three sections. The unit cells in the regions near the two ends (sections A and C) do not have any rotational offset to allow for a better mode coupling. In the central region of the rod (section B), which contains 32 unit cells, adjacent end-loaded dipole elements in the *z* direction were arranged to have a rotational shift of *φ*_rot_ = 15° in the *φ* direction. In this way, the capacitively coupled sub-wavelength unit cells of the twisted metasurface coating provide the power confinement, while the helical arrangement of the elements enables the desired broadband non-resonant circular birefringence. We emphasize that this approach is drastically different from those based on planar chiral metasurfaces, where the optical activity arises from strongly coupled transverse electric and/or magnetic resonators^54–56^. As displayed in the inset of Fig. 3a, the electric field distributions in the *x*-*y* plane at seven different positions along the axis of the rod unambiguously illustrate that the plane of polarization gradually rotates as the wave propagates in the *z* direction while maintaining a strong confinement. It is revealed that the achieved optical activity is a consequence of the accumulated small amount of polarization rotation enabled by the rotational shift of successive unit cells, which are coupled capacitively in a non-resonant manner. In particular, at 3.45 GHz, the polarization of the highly confined guided wave experiences a rotation of 90° after a propagation length of about 7*λ*_0_. It should be noted that the rotational shift parameter *φ*_rot_ can also be chosen to have other values (see Supplementary Fig. 7). As 8 unit cells were employed surrounding the Teflon rod, each one occupies a range of 45° in the *φ* direction. A small value of *φ*_rot_ indicates a minor twisting perturbation while a large value of *φ*_rot_, i.e., closer to 22.5°, leads to weaker coupling between successive unit cells, both of which result in a slow polarization rotation. As a special case, when *φ*_rot_ = 22.5°, i.e., half of the unit cell size in the *φ* direction, no polarization rotation is expected as the structure possesses a mirror symmetry. Simulations showed that the strongest optical activity takes place when *φ*_rot_ is around 11.25°, which provides a balance between twisting perturbation and inter-element coupling. Further studies have confirmed that this optical activity behavior is independent of the initial plane of the linearly polarized waves emitted from the probe, confirming that the observed polarization rotation is distinct from that reported previously in waveguides based on anisotropic phase shifting^57^. It should be noted that the low absolute transmission is caused by the bi-directional radiation of the dipole probes rather than the coated DW. The transmission (*T*_x_ and *T*_y_), defined by the ratio of the +*z* directional power flow inside the DW at the 4th and 37th unit cells, shows that the waveguide alone maintains high transmission properties (see Supplementary Fig. 8), where the effect of adding the twisting perturbation can also be clearly identified.

The planar twisted metasurface was fabricated and conformed to a Teflon rod, as shown in Fig. 1e. The measured and simulated co-polarized (*T*_xx_) and cross-polarized (*T*_yx_) transmission curves (Fig. 3b) differ from those of the coated waveguide with a preserved plane of polarization (Fig. 2f). More specifically, the amplitude of *T*_yx_ becomes significantly larger within a broad frequency range, implying that the optical activity arises from the twisting of the metasurface unit cells. The measured results closely match the simulation predictions, with a slight blue-shift due to fabrication and assembly inaccuracy (Fig. 3b). The dip observed in the *T*_yx_ curve indicates that a 90° polarization rotation occurs at around 3.66 GHz. To confirm the broadband optical activity, the state-of-polarization was further characterized by recording the transmitted power while rotating the receiving probe around the axis of the Teflon rod, i.e., the *z* axis. The measured state-of-polarization patterns of the coated waveguides with and without the twisting perturbation in the metasurface at a series of frequencies across the band of interest are presented in Fig. 3c. It can be seen that the plane of polarization of the guided wave propagating in the waveguide coated by the twisting metasurface has a frequency-dependent rotation, demonstrating the existence of broadband optical activity with a bandwidth of >50%. In contrast, no polarization rotation was observed in the waveguide with the non-twisting metasurface coating. The measured rotation angle (*θ*) and rotation angle per wavelength (*θ/λ*_0_) as a function of frequency are reported in Fig. 3d. In the frequency range of interest, the rotation angle monotonically grows as the frequency increases. This phenomenon is due to the fact that the twisting brings about more prominent circular birefringence at higher frequencies where a stronger mode confinement occurs. In particular, approaching the band edge, a saturation of the rotation power can be observed with a maximal *θ/λ*_0_ of around 13°.

### Low-scattering and optically active open waveguides

The metasurface guiding layer makes possible the transformative physical properties of power confinement and strong optical activity in a sub-wavelength cylindrical DW as demonstrated above. However, the collective electric response of the added structured surface significantly increases the scattering signature of the waveguide when illuminated by waves emanating from nearby radiating devices (Supplementary Fig. 9a), thus potentially causing signal blockage and/or a disturbance to neighboring systems operating in the same frequency range. Hence, it would be highly desirable to reduce the coated DWs’ scattering signature at the operational frequencies of neighboring systems while simultaneously maintaining their salient waveguiding properties. We emphasize that this is fundamentally different from previously demonstrated cloaks for antennas^50^, where the operational frequencies of the antenna and the cloaking coating are different. Here, the highly confined waveguiding and cloaking functionalities are achieved within the same frequency range, which represents an important practical advantage for crosstalk reduction in densely packed multi-waveguide systems.

**Fig. 4:**
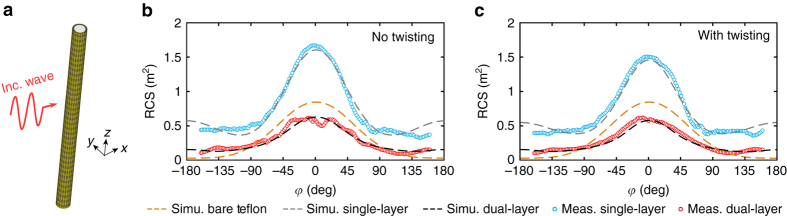
Scattering properties of the sub-wavelength dielectric waveguide with metasurface coatings. **a** Configuration of the finite-length coated Teflon rod waveguide excited by a TM polarized plane wave propagating in the +*x* direction. The simulated and measured RCS patterns in the *x*-*y* plane at the optimal frequency for the coated Teflon rod waveguide **b** without and **c** with twisting perturbation on the inner metasurface

For a long cylindrical structure, the scattering signature under a transverse magnetic (TM) wave excitation is most prominent and therefore will be considered here (Fig. 4a). To this end, the waveguiding and scattering properties of the dielectric rod waveguide with a dual-layer metasurface coating were investigated, with the goal of simultaneously achieving scattering reduction and strong sub-wavelength power confinement. Since most of the power of the HE_11_ mode guided wave is well confined within the rod, the outer cloaking layer would only have a minor impact as it modifies the field that is leaked outside the inner guiding layer (see Supplementary Note 2 and Supplementary Fig. 3). On the other hand, by solving the scattered field for the coated rod illuminated by a TM polarized plane wave, it was found that a weakly capacitive *Z*_szφ_ for the outer cloaking layer along with a sub-wavelength distance in between the two metasurface layers can greatly reduce the scattering signature by about 80%, as compared to that of the rod with only the inner guiding layer (see Supplementary Note 5). Remarkably, the outer cloaking layer with a weakly capacitive *Z*_szφ_ operates at frequencies far away from its own resonance, such that it helps to improve the power confinement of the confined HE_11_ mode without severely deteriorating the low-loss property of the coated waveguide. As shown in Fig. 1c, a metasurface comprised of a capacitively coupled end-loaded dipole array was adopted to form the outer cloaking layer, with 20 elements incorporated in the *φ* direction. Its capacitive inter-cell coupling is weaker than that of the inner guiding layer. The final waveguide, including two metasurface coating layers, has a radius *a*_c_ = 16.8 mm, which is less than 0.2*λ*_0_ in the frequency range of interest. As can be observed in Fig. 1c, the dispersion curve resembles that displayed in Fig. 1b with the only difference being a very slight red-shift in the band edge frequency of about 0.23 GHz, indicating well-maintained waveguiding properties with a mode area of 0.16$\lambda _0^2$ in the presence of the additional cloaking layer. The added coating only slightly increases the loss of the structure, yielding an attenuation constant *α*_z_ *p* with a value of less than 1.7 × 10^−3^ and a propagation length of more than 69*λ*_0_ in the frequency range of interest, i.e., the highlighted region in Fig. 1c (see Supplementary Note 3 and Supplementary Fig. 5).

**Fig. 5:**
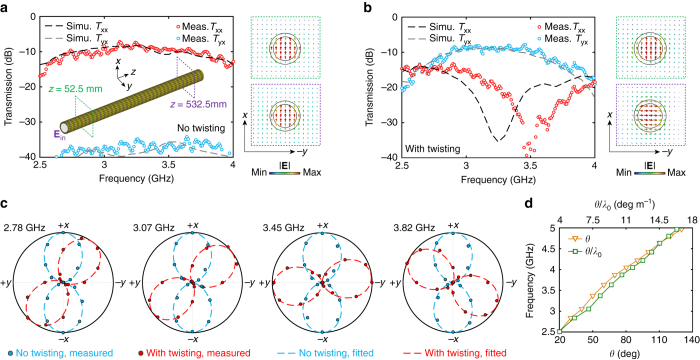
A sub-wavelength dielectric rod waveguide with dual-layer metasurface coatings. Simulated and measured co-polarized (*T*_xx_) and cross-polarized (*T*_yx_) transmission between the transmitting and receiving probes for the inner coating layer **a** without and **b** with twisting perturbation. The insets show the configuration and the snapshots of the vector electric field distribution at 3.3 GHz in the *x*-*y* planes at two different positions along the axis of the rod when excited by an *x* polarized probe. **c** Measured state-of-polarization patterns of the Teflon rod waveguide with a dual-layer coating for the inner metasurface layer with and without twisting perturbation. **d** Measured rotation angle and the rotation angle per wavelength as a function of frequency

The highly flexible outer metasurface cloaking layer was fabricated and assembled with the Teflon rod coated by the inner guiding layer (Fig. 1f). Two foam layers, each with a thickness of 2 mm, were used as spacers. It is important to note that the weight of the final dual-layer metasurface coated waveguide is almost the same as that of the original uncoated DW. For the case where the inner guiding layer contains the non-twisting metasurface, the linearly polarized guided mode is maintained with no polarization rotation as manifested by the huge difference between the measured co-polarized (*T*_xx_) and cross-polarized (*T*_yx_) transmission curves (Fig. 5a), which agrees well with the simulated results. The strength of the co-polarized transmission is slightly higher than that of the rod with a single-guiding layer coating, resulting from the stronger field confinement due to the added outer cloaking layer, especially at higher frequencies. In contrast, when the metasurface with a twisting perturbation of its unit cells was chosen as the inner guiding layer, significantly stronger measured cross-polarized transmission can be observed over a broad frequency range. This is due to the polarization rotation with a co-polarized transmission null occurring at around 3.5 GHz (Fig. 5b), indicating that the linearly polarized input wave has been completely rotated to its cross polarization state. The simulated field distributions at locations close to the input and output of the waveguide clearly show the polarization rotation phenomenon of the well-maintained HE_11_ mode for the case where the twisting metasurface guiding layer was employed (inset of Fig. 5a, b). The measured state-of-polarization patterns for the dual-layer metasurface coated waveguides are presented in Fig. 5c, while the measured rotation angle and the rotation angle per wavelength as a function of frequency are reported in Fig. 5d. A comparison between Figs. 5c and 3c clearly confirms that the outer cloaking layer has a minor impact on the guiding properties of the DW coated only by the inner guiding layer.

The radar cross section (RCS) of the finite-length Teflon rod waveguide with the single- and dual-layer coatings in the *x*–*y* plane were further characterized in an anechoic chamber using two horn antennas. It was found that the frequency of the lowest RCS occurs at 3.72 and 3.75 GHz, respectively, for the inner guiding layer without and with twisting perturbation, which is in good agreement with the simulations (Fig. 4b, c). For comparison purposes, the corresponding RCS pattern is also plotted for a bare Teflon rod with a radius of *a* = 16.8 mm. It is seen that with the outer cloaking layer, a significant suppression of RCS can be achieved at all observing angles, realizing a measured total RCS reduction of around 63%. The RCS of the proposed structure is comparable to that of the bare Teflon rod, while simultaneously possessing strong power confinement and optical activity. Importantly, the scattering reduction phenomenon is robust to the twisting of the unit cells in the inner metasurface guiding layer, which is an intriguing and potentially transformative property that has not been reported in previous scattering cancellation coatings^16–18^. It should be noted that by adding more layers, multispectral scattering reduction can be made possible while maintaining all the desired waveguiding properties and a similar waveguide cross section^58^.

### Broadband right-angle open waveguide bend

**Fig. 6:**
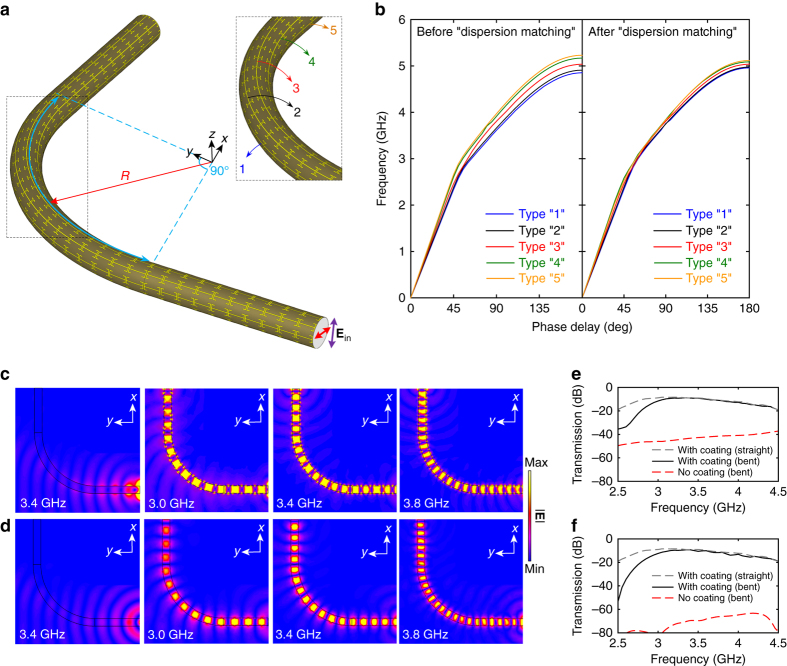
A right-angle dielectric rod waveguide bend with a single-layer metasurface coating. **a** Configuration of the right-angle Teflon rod waveguide bend coated by the metasurface guiding layer with an inhomogeneous distribution of unit cells in the bending section. The inset shows the enlarged view of the bending section, with the 5 types of unit cells labeled out. **b** Simulated dispersion curves for the five types of unit cells before and after “dispersion matching”. The dimensions for unit cells in progressive order from type “1” to type “5” are: *p* = 14, 14.3, 15, 15.7, 16 mm, *q* = 7.05, 6.75, 6.5, 6.2, 6 mm, and *s* = 0.35, 0.37, 0.5, 0.7, 0.75 mm. Snapshots of the electric field distribution of a bare and the coated Teflon rod waveguide bending in the **c** electric and **d** magnetic field plane. The simulated transmission between the transmitting and receiving probes for the bare and coated Teflon rod waveguide bending in the **e** electric and **f** magnetic field plane, as well as for the straight coated Teflon rod waveguide

In many systems, waveguide bends are unavoidable for routing the flow of signals with more degrees of freedom for enabling targeted functionalities and shrinking the overall device footprint. The capability demonstrated for the mode confinement in a straight configuration suggests that the proposed metasurface coating design methodology could potentially be extended to enable highly-efficient waveguiding in curved DWs, where severe wave leakage would occur with a sub-wavelength dielectric waveguide when the radius of curvature is on the order of less than a few wavelengths^59^. To investigate the efficacy for a curved dielectric rod waveguide, a right-angle bend with a bending radius of 191 mm, i.e., about 2*λ*_0_, was considered, which has the same overall length as that of the straight waveguides considered in the previous sections. In particular, for the coated waveguide, 20 unit cells were utilized in the bending section, while 10 unit cells were included in the straight sections on each of the two ends (Fig. 6a). As the inner and outer radii of the bending section are different, the unit cells that surround the rod were classified into five types, each of which has a different length (inset of Fig. 6a). Specifically, the type “1” and type “5” elements have the smallest and largest cell sizes, respectively, while the type “3” elements have the same cell size as those in the straight sections. Theoretically, such size differences may result in seriously mismatched wave momentum in the operational frequency range (Fig. 6b), leading to a degraded waveguiding performance due to the unequal phase delays across the cross section of the waveguide at the bending section (see Supplementary Fig. 10). In order to overcome this, judicious tailoring of the unit cell dimensions was carried out numerically (see Supplementary Table 1). As a result of the dispersion engineering, nearly identical dispersion curves were achieved for the five types of modified unit cells in the frequency range between 2.5 and 4.3 GHz (Fig. 6b).

The simulated electric field distributions between two linearly polarized dipole probes placed at the two ends of the waveguides are reported in Fig. 6c, d, for the rod bending in the electric and magnetic field plane, respectively. Without the coating, the DW exhibits poor power confinement such that the majority of the power coupled into the waveguide leaks out into free space at the bending. Such severe leakage can be alleviated by greatly increasing the bending radius, but at the expense of a much larger footprint and heavier weight. As predicted by the dispersion curves in Fig. 6b, the presence of the modified metasurface guiding coating enables smooth propagation of the strongly confined modes along the waveguide bend over a broad frequency range. In particular, Fig. 6e, f show that the coating greatly enhances the transmission by > 20 dB for the electric field plane bending case and > 50 dB for the magnetic field plane bending case. Remarkably, the transmission for the 90° waveguide bend with the dispersion-matched metasurface guiding coating is almost the same as that of the straight coated waveguide, which is also presented in Fig. 6e, f, further confirming the effectiveness of the proposed ultra-thin coating design modification for a curved configuration. Such broadband dispersion matching on a subwavelength scale can potentially be extended to the design of other metasurfaces for tailoring the flow of signals over a broad bandwidth.

## Discussion

In conclusion, we have proposed and demonstrated a fully conformal quasi-2D approach to achieving unprecedented control over various electromagnetic properties of a DW simultaneously by tailoring the tensorial surface impedances of flexible and low-loss metasurfaces. While the inner guiding layer enables a highly confined guided mode within a sub-wavelength cross section, the outer cloaking layer greatly suppresses the scattering signature. By introducing structural chirality into the inner metasurface layer without modifying the outer coating, it is possible to further achieve non-resonant broadband optical activity. Moreover, it was shown that the effectiveness of the artificial coating can be well maintained for waveguide bends by properly matching the dispersion properties of the metasurface unit cells. The demonstrated flexible coating represents previously unattainable capabilities for achieving simultaneous control over the propagation, polarization and scattering of electromagnetic waves. The presented design methodology brings about new and exciting possibilities of multifunctional wave manipulation by exploiting the unique surface electromagnetic properties of metasurfaces in a conformal platform. It is expected that the concept and associated design strategy can be further applied to achieve superior waveguiding components and other novel electromagnetic devices with multiple functionalities at terahertz frequencies and possibly even in the infrared regime.

## Methods

### Transmission and scattering cross section characterizations

The transmission amplitudes of the Teflon rod waveguide with and without a metasurface coating were measured in an anechoic chamber (see Supplementary Fig. 11). Two impedance-matched balanced linearly polarized dipole probes were employed. In the majority of the band of interest, the port reflection was maintained below −10 dB for the three cases of interest: a bare Teflon rod, the Teflon rod with a single-layer coating and the Teflon rod with a dual-layer coating. The RCS patterns in the horizontal *x*–*y* plane of the finite-length cylinders with and without the coatings were characterized in an anechoic chamber. Two double-ridged S-band horn antennas connected to a vector network analyzer were used—one with a fixed position as the transmitter and the other that was rotated around the cylinder in the horizontal plane as the receiver. Both horn antennas were 1.35 m (>10 *λ*_0_) away from the cylinder with their transmitted/received electric field polarized in the *z*-direction. Due to the finite size of the horn antennas, RCS information within the angular range of 160° ≤ *φ* ≤ 200° was not available. The complex transmission coeffcient, *S*_21b_(*f*, *φ*), between the two horn antennas without the presence of any object was recorded as the background signal. The scattering signal of the object, *S*_21s_(*f*, *φ*), was obtained by removing *S*_21b_(*f*, *φ*) from the complex transmission coefficient (*S*_21_(*f*, *φ*)) measured with the presence of the object as *S*_21s_(*f*, *φ*) = *S*_21_(*f*, *φ*)−*S*_21b_(*f, φ*). A post*-*processing gating in the time domain was applied to the measured signal as *S*_21sg_(*f*, *φ*) = DFT{W*(t*)•IDFT{*S*_21s_(*f*, *φ*)}}, where DFT and IDFT are the Discrete Fourier Transform and its Inverse, W(*t*) is a rec*t*angular window function used to gate out undesired mutipath signals, and the dot denotes a multiplication operation. The RCS was then calculated by using RCS(*f*, *φ*) = (4π)^3^ *R* ^4^ *|S*_21sg_(*f*, *φ*)|^2^/(*G*_t_ *G*_r_(*λ*_0_)^2^), where *R* is the distance between the target and the receiving/transmitting anatenna, while *G*_t_ and *G*_r_ are the absolute gains of the tranmitting and receiving antennas, respectively^60^.

### Data availability

The data that support the findings of this study are available from the corresponding author on request.

## Electronic supplementary material

Supplementary Information
