# Supplementary material for: Conformal metasurface-coated dielectric waveguides for highly confined broadband optical activity with simultaneous low-visibility and reduced crosstalk
Source: Nat Commun. 2017 Aug 25;8:356. doi: 10.1038/s41467-017-00391-0 (PMC5572855; doi:10.1038/s41467-017-00391-0)
Supplement: Supplementary file 1 — Supplementary Information [file 41467_2017_391_MOESM1_ESM.pdf]

Description of Supplementary Files

File Name: Supplementary Information
Description: Supplementary Figures, Supplementary Table, Supplementary Notes and Supplementary References

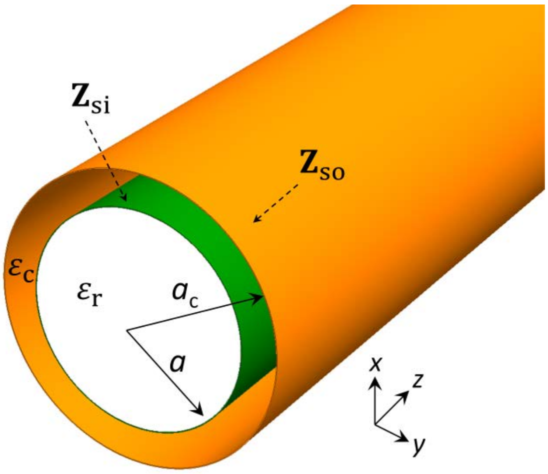

Supplementary Figure 1. Schematics of a dielectric waveguide coated by homogeneous impedance surfaces. A dielectric rod waveguide, with a radius of a and a relative permittivity of ε_r, is coated by two layers of tensorial impedance surfaces with a homogeneous surface impedance tensor of $\mathbf{Z}_{si}$ and $\mathbf{Z}_{so}$, respectively. The radius of the outer impedance surface is a_c. The spacer in between the two impedance surfaces has a relative permittivity of ε_c.

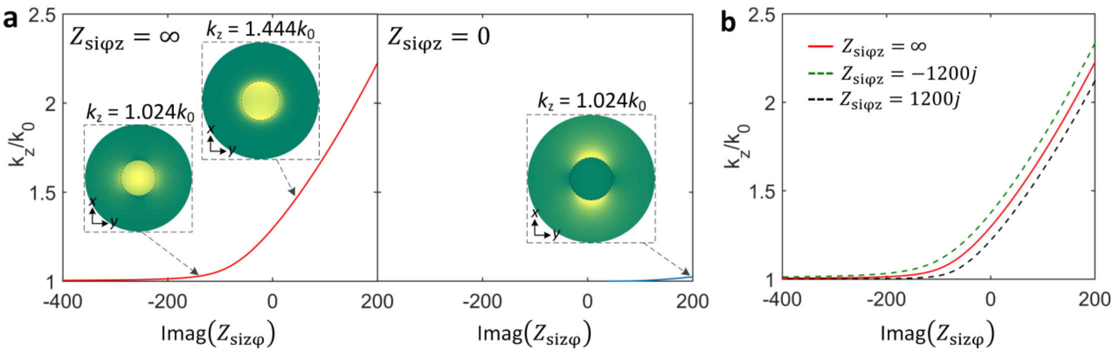

Supplementary Figure 2. Propagation constant of the HE_{11} mode of a dielectric waveguide coated by a single-layer metasurface. (a) The calculated normalized propagation constant (k_z/k_0) of the HE_{11} mode as a function of $Z_{\mathrm{siz\varphi}}$ for the cases where $Z_{\mathrm{si\varphi z}} = 0$ (short circuit) and $Z_{\mathrm{si\varphi z}} = \infty$ (open circuit), respectively. The insets show the normalized power distributions in the *x*-*y* plane for selected values of $Z_{\mathrm{siz\varphi}}$ (max: yellow, min: green). **(b)** The calculated normalized propagation constant (k_z/k_0) of the HE_{11} mode as a function of $Z_{\mathrm{siz\varphi}}$ for the cases where $Z_{\mathrm{si\varphi z}} = \infty$ (open), $Z_{\mathrm{si\varphi z}} = -1200j$ (capacitive), and $Z_{\mathrm{si\varphi z}} = 1200j$ (inductive).

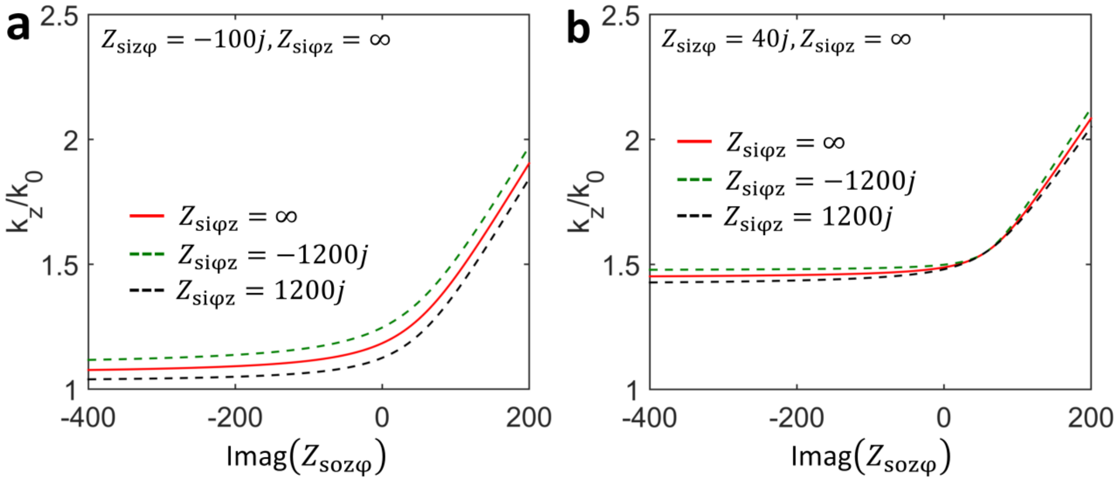

Supplementary Figure 3. Propagation constant of the HE$_{11}$ mode of dielectric waveguides coated by a dual-layer metasurface. The calculated normalized propagation constant (k_z/k_0) of the HE$_{11}$ mode as a function of $Z_{\mathrm{soz\varphi}}$ for the cases where where $Z_{\mathrm{so\varphi z}} = \infty$ (open circuit), $Z_{\mathrm{so\varphi z}} = -1200j$ (capacitive), and $Z_{\mathrm{so\varphi z}} = 1200j$ (inductive). The properties of the lossless inner tensorial impedance surface are **(a)** $Z_{\mathrm{siz\varphi}} = -100j$, $Z_{\mathrm{si\varphi z}} = \infty$ (open circuit), and **(b)** $Z_{\mathrm{siz\varphi}} = 40j$, $Z_{\mathrm{si\varphi z}} = \infty$ (open circuit).

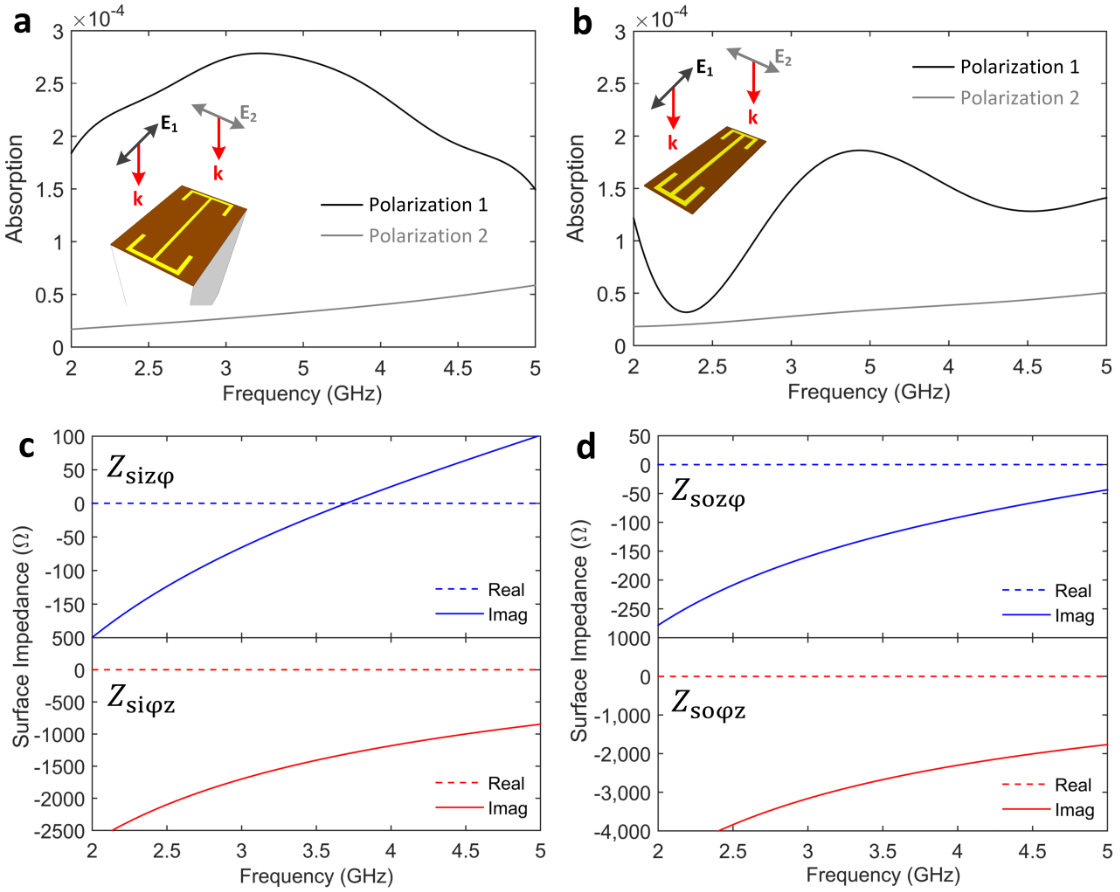

Supplementary Figure 4. Electromagnetic properties of the metasurface coating. Simulated absorption loss of **(a)** the inner metasurface guiding layer and **(b)** the outer metasurface cloaking layer when excited by incident plane waves with different polarizations shown in the inset. Polarization 1: electric field parallel to the central bar of the metasurface resonator. Polarization 2: electric field perpendicular to the central bar of the metasurface resonator. The finite conductivity of copper (σ = 5.8e7 S m^{-1}) and the permittivity of the Taconic TLY-5 substrate (ε_r = 2.2, tanδ = 0.0009) were taken into account in the simulations. For all cases, the absorption loss is extremely low. **(c)** The retrieved effective $Z_{siz\varphi}$ and $Z_{si\varphi z}$ for the inner metasurface guiding layer. **(d)** The retrieved effective $Z_{soz\varphi}$ and $Z_{so\varphi z}$ for the outer metasurface cloaking layer.

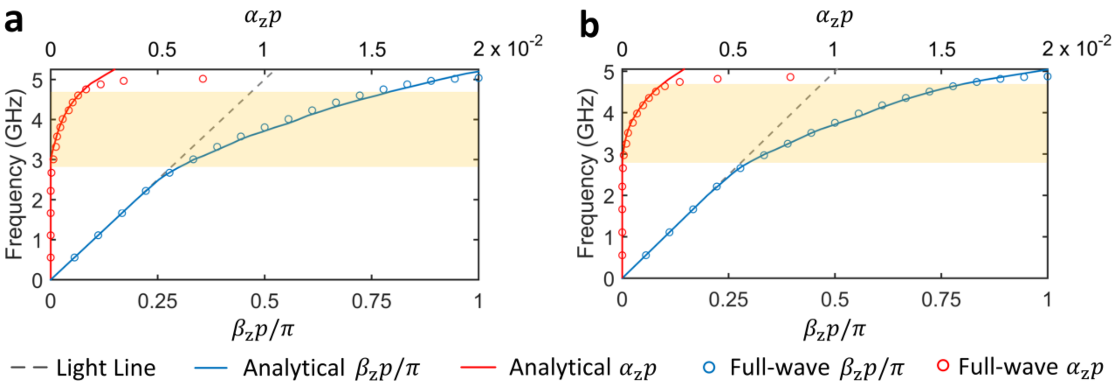

Supplementary Figure 5. Dispersion curves of the HE_{11} mode of metasurface-coated dielectric waveguide. Analytically calculated and full-wave simulated dispersion curves of β_z and α_z corresponding to the HE_{11} mode for the Teflon waveguide with **(a)** the metasurface guiding layer and **(b)** the metasurface guiding and cloaking layers. The highlighted region is from 2.8 to 4.59 GHz.

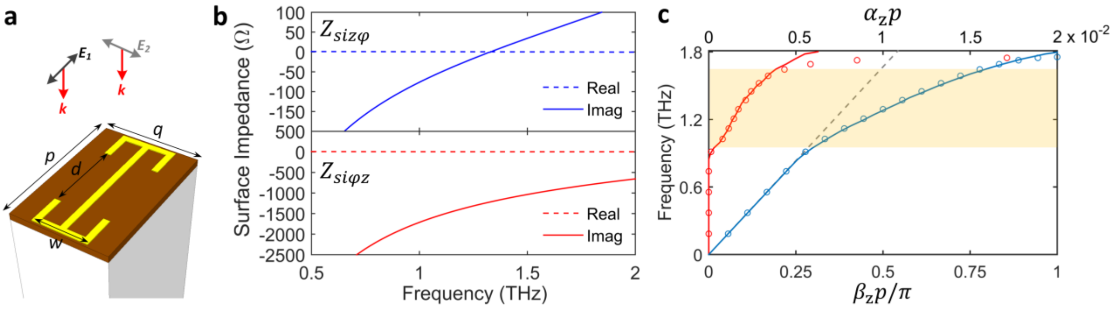

Supplementary Figure 6. Metasurface coating design for a dielectric waveguide at terahertz frequencies. (a) Geometry of the metasurface guiding layer excited by incident plane waves with different polarizations at terahertz frequencies. The dimensions are: p = 45, q = 29.8, d = 23.5, w = 17, all in μm. The width of all the metallic traces is 2 μm. **(b)** The retrieved effective $Z_{\mathrm{siz\varphi}}$ and $Z_{\mathrm{si\varphi z}}$ for the metasurface guiding layer. **(c)** Analytically calculated and full-wave simulated dispersion curves of β_z and α_z corresponding to the HE_{11} mode for the HDPE (ε_r = 2.088, tanδ = 0.0034) terahertz waveguide with a radius of 36 μm coated by the metasurface guiding layer. Grey dashed line: light line of free space. Red line: analytical $\alpha_z p$. Red dots: numerical $\alpha_z p$. Blue line: analytical $\beta_z p$. Blue dots: numerical $\beta_z p$. The highlighted region is from 0.95 to 1.65 THz.

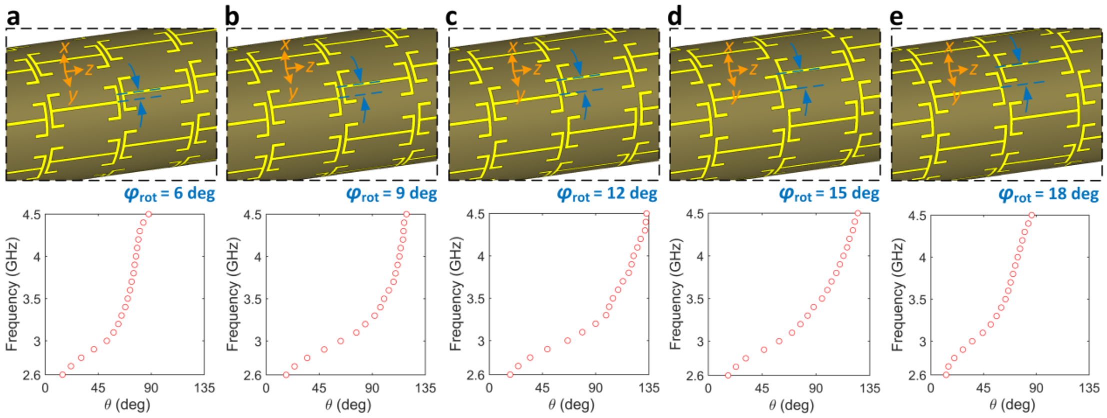

Supplementary Figure 7. A **dielectric waveguide coated by a twisting metasurface.** Geometry and simulated polarization rotation angle (θ) as a function of frequency for the Teflon rod waveguide coated by the metasurface guiding layer with **(a)** φ_{rot} = 6°, **(b)** φ_{rot} = 9°, **(c)** φ_{rot} = 12°, **(d)** φ_{rot} = 15°, and **(e)** φ_{rot} = 18°. The blue arrows define the rotational offset φ_{rot} in between the center bars (blue dashed lines) of adjacent unit cells.

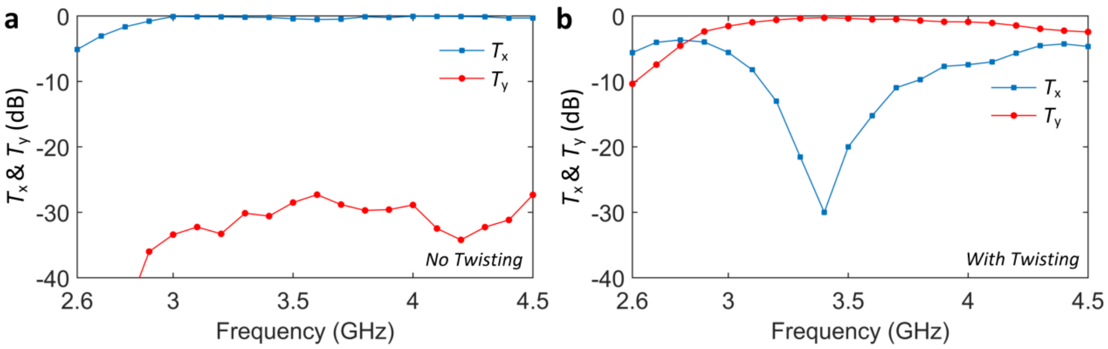

Supplementary Figure 8. Transmission of the metasurface coated dielectric waveguides. Transmission (T_x and T_y) for the dielectric rod waveguide coated by the metasurface guiding layer **(a)** without and **(b)** with the twisting perturbation in the unit cells, defined by the ratio of the +*z* directional power flow inside the dielectric waveguide at the 4th and 37th unit cells, which are close to the input and output ends of the coated dielectric waveguide. Specifically, $T_x = \iint_{S_{37th}} Re[E_x \times H_y^*] / \iint_{S_{4th}} Re[E_x \times H_y^*]$, while $T_y = \iint_{S_{37th}} Re[-E_y \times H_x^*] / \iint_{S_{4th}} Re[E_x \times H_y^*]$.

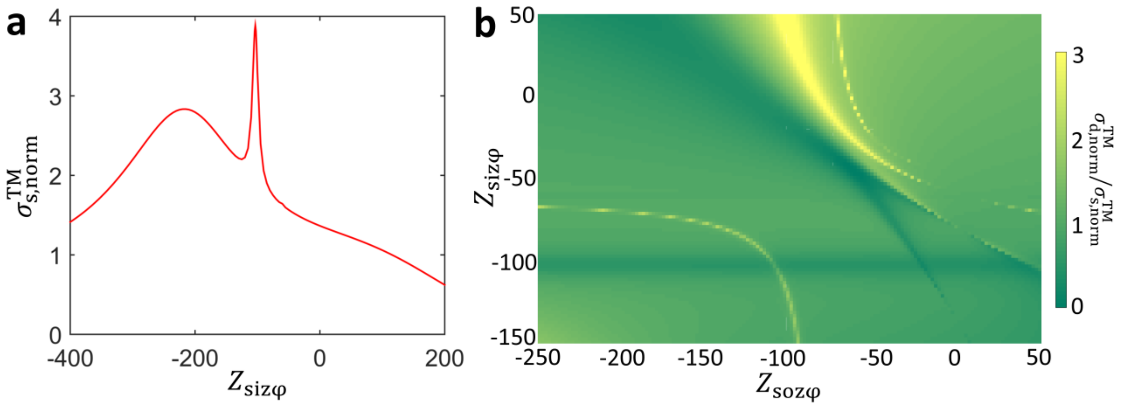

Supplementary Figure 9. Scattering properties of a metasurface-coated dielectric waveguide. (a) The calculated normalized scattering width ($\sigma^{\mathrm{TM}}_{\mathrm{s,norm}}$) as a function of $Z_{\mathrm{siz\varphi}}$ for the case of a Teflon rod, with a = $0.144\lambda_0$, coated by the metasurface guiding layer. **(b)** Contour plot of the calculated scattering width ratio between a Teflon rod coated by the metasurface guiding and cloaking layers and the same Teflon rod coated by the metasurface guiding layer ($\sigma^{\mathrm{TM}}_{\mathrm{d,norm}}/\sigma^{\mathrm{TM}}_{\mathrm{s,norm}}$) as a function of $Z_{\mathrm{siz\varphi}}$ and $Z_{\mathrm{siz\varphi}}$, with a = $0.144\lambda_0$ and a_{c} = $0.189\lambda_0$.

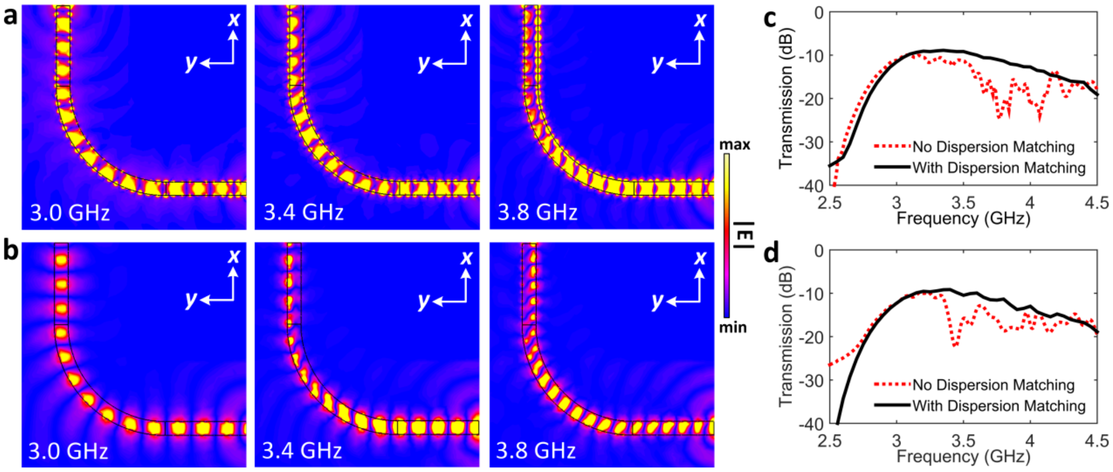

Supplementary Figure 10. A right-angle dielectric waveguide bend coated by a single-layer metasurface without dispersion matching. Snapshots of the electric field distribution of a bare Teflon rod waveguide bend and the coated Teflon rod waveguide bend in (**a**) the electric and (**b**) magnetic field plane. The simulated transmission between the transmitting and receiving probes for the bare Teflon rod waveguide bend and the coated Teflon rod waveguide bend in (**c**) the electric and (**d**) magnetic field plane.

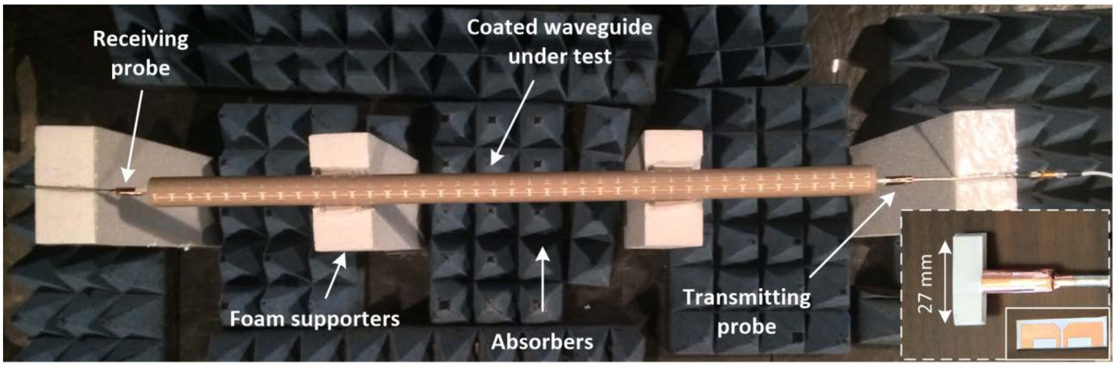

Supplementary Figure 11. Measurement setup. Transmission measurement setup for testing the metasurface coated Teflon rod waveguide. The inset shows a photograph of the employed impedance-matched balanced dipole probe.

**Type**	***p*** (mm)	***q*** (mm)	***s*** (mm)
1	14.0	7.05	0.35
2	14.3	6.75	0.37
3	15.0	6.5	0.5
4	15.7	6.2	0.7
5	16.0	6	0.75

Supplementary Table 1. Geometrical dimensions for the five types of unit cells in the bending section. The parameter p and q refer to the periodicity and width of the metasurface unit cell, while s refers to the spacing in between adjacent unit cells.

Supplementary Note 1: Analytical solution of the HE_{11} mode of a dielectric rod waveguide coated by two concentric tensorial impedance surfaces

As shown in Supplementary Fig. 1, we consider a cylindrical dielectric rod waveguide with a radius of a and a relative permittivity of ε_r, that has its axis oriented along the z direction. It is coated by two tensorial impedance surfaces with homogeneous surface impedance tensors of $\mathbf{Z}_{\mathrm{si}}$ and $\mathbf{Z}_{\mathrm{so}}$ for the inside and outside layers respectively. For both layers, the surface impedance tensor has non-zero values only for the off-diagonal elements, which may be defined as

$$\begin{bmatrix} E_{\varphi} \\ E_{\mathrm{z}} \end{bmatrix} = \mathbf{Z}_{\mathrm{si(o)}} \begin{bmatrix} H_{\varphi} \\ H_{\mathrm{z}} \end{bmatrix} = \begin{bmatrix} 0 & Z_{\mathrm{si(o)\varphi z}} \\ Z_{\mathrm{si(o)z\varphi}} & 0 \end{bmatrix} \begin{bmatrix} H_{\varphi} \\ H_{\mathrm{z}} \end{bmatrix}. \tag{1}$$

For a general lossy impedance surfaces, the values of $Z_{\mathrm{si(o)z\varphi}}$ and $Z_{\mathrm{si(o)\varphi z}}$ are complex numbers, where the real parts represent loss. The inner layer is located at radius a while the outer layer is placed at radius a_c. The spacer in between the two coating layers has a relative permittivity of ε_c. The fundamental guided mode is the HE_{11} mode, which does not have a cutoff frequency. In order to calculate the dispersion curve and the field distribution of this mode for such a coated dielectric rod waveguide, the following steps are employed [1]. First, the z components of the electric and magnetic fields in different regions are derived. For the region inside the central dielectric rod, the electric and magnetic fields (E_{1z} and H_{1z}) are expressed as

$$E_{1\mathrm{z}} = A_1 J_1(k_{1\rho}\rho)\cos(\varphi)e^{-jk_{\mathrm{z}}z}, \tag{2}$$

$$H_{1\mathrm{z}} = B_1 J_1(k_{1\rho}\rho)\sin(\varphi)e^{-jk_{\mathrm{z}}z}. \tag{3}$$

For the region in between the two tensorial impedance surface coatings, the electric and magnetic fields (E_{cz} and H_{cz}) are written as

$$E_{\mathrm{cz}} = [E_1 J_1(k_{\mathrm{c}\rho}\rho) + F_1 Y_1(k_{\mathrm{c}\rho}\rho)]\cos(\varphi)e^{-jk_{\mathrm{z}}z}, \tag{4}$$

$$H_{\mathrm{cz}} = [G_1 J_1(k_{\mathrm{c}\rho}\rho) + H_1 Y_1(k_{\mathrm{c}\rho}\rho)]\sin(\varphi)e^{-jk_{\mathrm{z}}z}. \tag{5}$$

Finally, for the free space region outside the outer coating layer, the electric and magnetic fields (E_{0z} and H_{0z}) can be formulated as

$$E_{0\mathrm{z}} = C_1 H_1^{(2)}(k_{0\rho}\rho)\cos(\varphi)e^{-jk_{\mathrm{z}}z}, \tag{6}$$

$$H_{0\mathrm{z}} = D_1 H_1^{(2)}(k_{0\rho}\rho)\sin(\varphi)e^{-jk_{\mathrm{z}}z}. \tag{7}$$

In Supplementary Equation 2 to Supplementary Equation 7, k_{z} represents the longitudinal wave number. $k_{1\rho}$, $k_{\mathrm{c}\rho}$, and $k_{0\rho}$ are the radial wave numbers in the three regions, respectively. A_1, B_1, C_1, D_1, E_1, F_1, G_1, and H_1 are unknown amplitude coefficients. The radial and longitudinal wavenumbers in the different regions must satisfy the respective dispersion relations which are expressed as

$$k_{1\rho}^2 + k_{\mathrm{z}}^2 = k_0^2\varepsilon_{\mathrm{r}}, \tag{8}$$

$$k_{\mathrm{c}\rho}^2 + k_{\mathrm{z}}^2 = k_0^2\varepsilon_{\mathrm{c}}, \tag{9}$$

$$k_{0\rho}^2 + k_{\mathrm{z}}^2 = k_0^2. \tag{10}$$

J_1 and Y_1 are the 1st order cylindrical Bessel functions of the first and second kind, and $H_1^{(2)}$ is the 1st order cylindrical Hankel function of the second kind. Based on these expressions for the *z* component of the electric and magnetic fields, the *ρ* and *φ* components of the electric and magnetic fields in all the regions can be readily obtained from Maxwell's equations. To solve for the unknown coefficients, the boundary conditions at the inner and outer tensorial impedance surfaces must be satisfied [2], which are given by

$$(E_{\mathrm{cz}} - E_{\mathrm{1z}})|_{\rho=a} = 0, \tag{11}$$

$$\left(E_{\mathrm{c\varphi}} - E_{1\mathrm{\varphi}}\right)|_{\rho=a} = 0, \tag{12}$$

$$2Z_{\mathrm{siz\varphi}}\left(H_{\mathrm{c\varphi}} - H_{1\mathrm{\varphi}}\right)|_{\rho=a} = (E_{\mathrm{1z}} + E_{\mathrm{cz}})|_{\rho=a}, \tag{13}$$

$$2Z_{\mathrm{si\varphi z}}(H_{\mathrm{cz}} - H_{\mathrm{1z}})|_{\rho=a} = \left(E_{1\mathrm{\varphi}} + E_{\mathrm{c\varphi}}\right)|_{\rho=a}, \tag{14}$$

and

$$(E_{\mathrm{0z}} - E_{\mathrm{cz}})|_{\rho=a_{\mathrm{c}}} = 0, \tag{15}$$

$$\left(E_{0\mathrm{\varphi}} - E_{\mathrm{c\varphi}}\right)|_{\rho=a_{\mathrm{c}}} = 0, \tag{16}$$

$$2Z_{\mathrm{soz\varphi}}\left(H_{0\mathrm{\varphi}} - H_{\mathrm{c\varphi}}\right)|_{\rho=a_{\mathrm{c}}} = (E_{\mathrm{0z}} + E_{\mathrm{cz}})|_{\rho=a_{\mathrm{c}}}, \tag{17}$$

$$2Z_{\mathrm{so\varphi z}}(H_{\mathrm{0z}} - H_{\mathrm{cz}})|_{\rho=a_{\mathrm{c}}} = \left(E_{0\mathrm{\varphi}} + E_{\mathrm{c\varphi}}\right)|_{\rho=a_{\mathrm{c}}}. \tag{18}$$

By inserting the field equations (Supplementary Equations 2 to 7) into Supplementary Equations 11 to 18, the propagation constant $k_{\mathrm{z}} = \beta_{\mathrm{z}} - j\alpha_{\mathrm{z}}$ and the field expressions can be obtained after some algebraic manipulation.

Supplementary Note 2: HE_{11} mode of a dielectric rod waveguide coated by lossless tensorial impedance surfaces

For a Teflon rod with a radius of a = 0.144λ_0 that is coated by a single lossless tensorial impedance surface, the dependence of the propagation constant of the HE_{11} mode on the value of $Z_{\mathrm{siz\varphi}}$ is shown in Supplementary Fig. 2a. It can be seen that when $Z_{\mathrm{si\varphi z}} = \infty$, *i.e.* an open circuit where the *z* component of the magnetic field is continuous across the boundary, a real number solution of the propagation constant can be obtained for a value of $Z_{\mathrm{siz\varphi}}$ within a range from -400j to 200j. Conversely, when $Z_{\mathrm{si\varphi z}} = 0$, *i.e.* a short circuit where the φ component of the electric field is forced to be zero at the boundary, a real number solution of the propagation constant exists only when $Z_{\mathrm{siz\varphi}}$ has an inductive value. Importantly, from the power distribution plots of the HE_{11} mode shown in the insets of Supplementary Fig. 2a, it can be seen that when $Z_{\mathrm{si\varphi z}} = \infty$, it is possible to obtain strongly confined power within the rod. Remarkably, for an inductive surface impedance value of $Z_{\mathrm{siz\varphi}} = 40j$, a propagation constant of 1.444k_0 is achieved, resulting in a mode area of only 0.13λ_0^2. For the case where $Z_{\mathrm{siz\varphi}} = -140j$, the majority of the power is still maintained within the rod even with k_z = 1.024k_0 and an accompanying mode area of 2.58λ_0^2, which is drastically different from that of a bare Teflon rod (see Fig. 1a in the main text). In contrast, when $Z_{\mathrm{si\varphi z}} = 0$, for a case where the same value of k_z = 1.024k_0 is maintained, almost all the power is located outside the rod near the surface with a much larger mode area of 3.43λ_0^2. This comparison indicates that by custom engineering the electromagnetic properties at the boundary of the dielectric rod waveguide, dramatically different power distributions for the HE_{11} mode can be realized. Importantly, the tensorial impedance surface can be employed to achieve sub-wavelength power confinement within a dielectric rod waveguide with a low permittivity.

The first case where $Z_{\mathrm{si\varphi z}} = \infty$ is the most interesting and useful one since the phenomenon of a greatly improved power confinement is insensitive to the variation of $Z_{\mathrm{siz\varphi}}$, implying that such an effect can be obtained over a broad frequency range. The range over which $Z_{\mathrm{siz\varphi}}$ transitions from capacitive to inductive can be utilized and realized by a series *LC* resonance in the *z* direction. Further studies indicate that the guiding property remains robust even when the value of $Z_{\mathrm{si\varphi z}}$ deviates from the ideal open circuit condition (see Supplementary Fig. 2b). The $Z_{\mathrm{si\varphi z}} = -1200j$ case is more favourable since it indicates a weakly capacitive property, which can be tailored to exhibit very broadband response as compared to the $Z_{\mathrm{si\varphi z}} = 1200j$ case where a strong inductive response is required.

When the second lossless tensorial impedance surface layer is added at an outer radius a_c, the properties of the HE_{11} mode is affected due to the fact that it changes the fields that are leaked outside the inner layer. However, when most of the power is well confined to within the rod due to the inner tensorial impedance surface, the effects of adding an outer impedance surface will be minor. Supplementary Fig. 3a,b show the impact of the outer impedance surface on the propagation constant of the HE_{11} mode, where the properties of the inner surface coating are $Z_{\mathrm{siz\varphi}} = -100j$, $Z_{\mathrm{si\varphi z}} = \infty$, and $Z_{\mathrm{siz\varphi}} = 40j$, $Z_{\mathrm{si\varphi z}} = \infty$, respectively. The spacer in between the two layers was considered to be foam which has a relative permittivity of 1.06 and a_c was chosen to be 0.189λ_0. It can be seen that for capacitive values of $Z_{\mathrm{soz\varphi}}$, the impact on the propagation constant is negligible. However, when $Z_{\mathrm{soz\varphi}}$ changes from capacitive into inductive, a more dramatic increase in the propagation constant can be observed. In addition, the guiding property is robust when the value of $Z_{\mathrm{so\varphi z}}$ deviates from the ideal open circuit condition. Finally, a weakly capacitive $Z_{\mathrm{so\varphi z}}$ response is favourable due to its inherent broadband property.

Supplementary Note 3: Impact of loss in the tensorial impedance surfaces on the propagation constant of the HE_{11} mode

In realistic scenarios, the conformal tensorial impedance surface coatings are implemented using metallic patterns printed on flexible dielectric substrates. The metals have a finite conductivity while the dielectric substrates have a non-zero loss tangent, resulting in a lossy coating. As a result, the propagation constant k_{z} is a complex number. By using the retrieved complex effective $Z_{\mathrm{si(o)z\varphi}}$ and $Z_{\mathrm{si(o)\varphi z}}$ for the inner (outer) metasurface coating layer, as presented in Supplementary Fig. 4c,d, the eigenmode problem was resolved using Muller's method to obtain the complex propagation constant ($k_{\mathrm{z}} = \beta_{\mathrm{z}} - j\alpha_{\mathrm{z}}$) of the strongly-confined HE_{11} mode in the proposed coated waveguide. To confirm the validity of the results obtained from the analytical model, eigenmode analysis was performed using full-wave electromagnetic solvers, where the loss can be retrieved from the complex eigen-frequencies [3].

As reported in Supplementary Fig. 5a, for the case where only the metasurface guiding layer was included, the dispersion curve of β_{z} is almost the same as that of the case with a lossless coating. The analytically calculated results agree well with those predicted by the full-wave solvers, except for some discrepancies at frequencies near the band edge. The imaginary part $\alpha_{\mathrm{z}}p$ is very small, which is less than 1.4×10^{-3} in the band of interest, *i.e.* the highlighted region. Such a small field attenuation factor indicates that the propagation length is about $424\lambda_0$ at 3.4 GHz, where the propagation length is defined by the distance over which the intensity of the guided mode is reduced by a factor of 1/*e*. For comparison, the dispersion curves of β_{z} and α_{z} of the HE_{11} mode for the Teflon waveguide with both the metasurface guiding and cloaking layers are shown plotted in Supplementary Fig. 5b. It can be seen that the results are very similar to those for the case where only the inner metasurface guiding layer was considered. In terms of loss, since the added outer metasurface cloaking layer is extremely low-loss and operates at frequencies far away from its resonance, it has almost no impact on the performance of $\alpha_{\mathrm{z}}p$ which remains less than 1.7×10^{-3} in the band of interest, *i.e.* the highlighted region. For comparison, at 3.4 GHz, the propagation length is slightly reduced to a value of about $383\lambda_0$. It should be noted that the propagation length values for the waveguide with a single-layer or dual-layer coating are larger than those based on strongly confined surface modes, such as spoof SPP waveguides [4].

Supplementary Note 4: **Conformal metasurface coating at terahertz frequencies**

To investigate the possibility of extending this technology into much higher frequencies, a conformal metasurface coating for a dielectric rod waveguide operating at terahertz frequencies was considered first. The unit cell is depicted in Supplementary Fig. 6a. Its metallic patterns, comprised of 200 nm thick gold, were printed on a 2 μm thick polyethylene naphthalate (PEN) substrate (ε_r = 2.56, tan δ = 0.003) [5]. The conductivity of gold was set to be 4.09×10^7 S m^{-1} [6]. The dielectric material of the rod waveguide was assigned to be high-density polyethylene (HDPE) (ε_r = 2.088, tan δ = 0.0034), which is extremely low-loss at terahertz frequencies [7]. The absorption loss of the unit cell when illuminated by plane waves with different polarizations is larger than that of the unit cell operating at microwave frequencies, it is still smaller than 0.5% in the majority of the frequency range of interest.

Considering a HDPE rod waveguide with a radius of 36 μm coated by a conformal metasurface with 8 unit cells in the φ direction, the dispersion curve of the HE_{11} mode was analytically calculated based on the retrieved dispersive effective $Z_{\mathrm{siz\varphi}}$ and $Z_{\mathrm{si\varphi z}}$ for the metasurface guiding layer (see Supplementary Fig. 6b). As shown in Supplementary Fig. 6c, it can be observed that the phase constant ($\beta_z p/\pi$) possesses a line shape similar to its microwave counterpart, with a simulated band edge at about 1.75 THz. In the frequency range of interest, *i.e.* the highlighted region, the analytical result agrees well with the numerically evaluated dispersion curve for the phase constant considering the realistic metasurface structure, again confirming that the discrete metasurface well approximates a homogeneous tensorial impedance surface. The imaginary part $\alpha_z p$ remains quite small such that it is less than 3.4×10^{-3} over the majority of the band of interest, *i.e.* the highlighted region. The increased loss comes from the higher loss in the dielectric materials and the smaller conductivity of gold in the terahertz regime as compared to that of copper at microwave frequencies. As an example, at 1.2 THz, the attenuation constant $\alpha_z p$ equals to 1.38×10^{-3}, implying a propagation length of $66\lambda_0$, which is superior to previously proposed terahertz sub-wavelength waveguides employing gap magnetic plasmon polaritons [8]. It should be noted that other technological challenges, such as fabrication and experimental setup, will need to be overcome before this technology can be successfully transitioned for terahertz applications.

Supplementary Note 5: Scattering properties of a dielectric rod waveguide coated by two concentric tensorial impedance surfaces

In addition to modifying the property of the guided HE_{11} mode in a dielectric rod waveguide, a conformal tensorial impedance surface coating can also be used to manipulate the scattering signature of the open waveguide. Following the analytical equations developed in Ref. [9], the scattered fields can be calculated, from which the scattering width can be obtained. For a long cylindrical structure, the scattering signature under a transverse magnetic (TM) wave excitation is the most prominent and thus is examined here. First, we consider the Teflon rod, with a radius of $a = 0.144\lambda_0$, coated by a single tensorial impedance surface. The scattering width ($\sigma_{\mathrm{s,norm.}}^{\mathrm{TM}}$) normalized to the wavelength in free space as a function of $Z_{\mathrm{siz\varphi}}$ is displayed in Supplementary Fig. 9a, where the value of $Z_{\mathrm{siz\varphi}}$ is limited to the range within which the desired waveguiding property is achieved, as discussed in Supplementary Note 1. It can be seen that, due to the added tensorial impedance surface, the normalized scattering width is significantly increased when compared to that of a bare Teflon rod with the same radius (which equals a value of 0.32). With the dual-layer tensorial impedance surface coating, scattering signature suppression can be achieved when $Z_{\mathrm{siz\varphi}}$ and $Z_{\mathrm{soz\varphi}}$ are chosen to be certain specific values, as indicated in Supplementary Fig. 9b. It is seen that even a scattering width with a value of nearly zero can be obtained. The values of $Z_{\mathrm{si\varphi z}}$ and $Z_{\mathrm{so\varphi z}}$ are not taken into account here because they would not affect the scattering width of the coated rod under a TM excitation at normal incidence. Further studies show that, with the optimal values of $Z_{\mathrm{siz\varphi}}$ and $Z_{\mathrm{soz\varphi}}$, for oblique incidence with angles smaller than 20°, a scattering reduction of more than 80% can still be achieved. This study provides the first demonstration that a dual-layer tensorial impedance surface coating can simultaneously control the waveguiding and scattering properties of a dielectric rod waveguide to achieve sub-wavelength power confinement of the HE_{11} mode and a greatly reduced scattering signature in a specified frequency range.

Supplementary References

1. Balanis, C. A. *Advanced Engineering Electromagnetics* (Wiley, 1989).
2. Senior, T. B. A. & Volakis, J. L. *Approximate Boundary Conditions in Electromagnetics* (IEE Press, 1995).
3. Wan, X. & Cui, T. J. Guiding spoof surface plasmon polaritons by infinitely thin grooved metal strip. *AIP Adv.* **4**, 047137 (2014).
4. Shen, X., Cui, T. J., Martin-Cano, D. & Garcia-Vidal, F. J., Conformal surface plasmons propagating on ultrathin and flexible films. *Proc. Natl. Acad. Sci. USA* **110**, 40-45 (2013).
5. Walia, S. *et al.* Flexible metasurfaces and metamaterials: A review of materials and fabrication processes at micro- and nano-scales. *Appl. Phys. Rev.* **2**, 011303 (2015).
6. Lee, S. *et al.* Reversible stretchable and tunable terahertz metamaterials with wrinkled layouts. *Adv. Mater.* **24**, 3491-3497 (2012).
7. Atakaramians, S., Afshar, S. V., Monro, T. M. & Abbott, D. Terahertz dielectric waveguides. *Adv. Opt. Photon.* **5**, 169-215 (2013).
8. Ishikawa, A., Zhang, S., Genov, D. A., Bartal, G. & Zhang, X. Deep subwavelength terahertz waveguides using gap magnetic plasmon. *Phys. Rev. Lett.* **102**, 043904 (2009).
9. Jiang, Z. H. & Werner, D. H. Dispersion engineering of metasurfaces for dual-frequency quasi-three-dimensional cloaking of microwave radiators. *Opt. Express* **24**, 9629-9644 (2016).
